# Radiomics: Assessing Significance and Correlation with Ground-Truth Data in Precision Medicine in Lung Adenocarcinoma

**DOI:** 10.3390/bioengineering12060576

**Published:** 2025-05-27

**Authors:** Rama Vasantha Adiraju, Kapula Kalyani, Gunnam Suryanarayana, Mohammed Zakariah, Abdulaziz S. Almazyad

**Affiliations:** 1 Department of Electronics and Communication Engineering, Aditya University, Surampalem 533437, Andhra Pradesh, India; vasantha.adiraju@acet.ac.in (R.V.A.); kalyani.kapula@acet.ac.in (K.K.); 2 Department of Electronics and Communication Engineering, Siddhartha Academy of Higher Education (Deemed to be a University), Vijayawada 520007, Andhra Pradesh, India; gsuryanarayana@vrsiddhartha.ac.in; 3 Department of Computer Science and Engineering, College of Applied Studies and Community Service, King Saud University, P.O. Box 22459, Riyadh 11495, Saudi Arabia; 4 Department of Computer Engineering, College of Computer and Information Sciences, King Saud University, Riyadh 11543, Saudi Arabia; mazyad@ksu.edu.sa

**Keywords:** radiomics, lung adenocarcinoma, random walk, ensemble segmentation, Spearman’s correlation coefficient

## Abstract

Radiomics, an emerging discipline integrating imaging science, computational biology, and clinical oncology, enables the extraction of quantitative biomarkers from medical images for improved diagnosis and prognosis. However, variability in imaging protocols and insufficient validation studies hinder the clinical reliability of these biomarkers, limiting their integration into precision medicine. This study addresses these challenges by proposing an RW-ensemble method for extracting and validating radiomic features from segmented lung nodules. Using the Lung CT-Diagnosis dataset, which comprises CT images of 61 patients with segmentation annotations, nearly 38 radiomic features were extracted, incorporating texture-based features from the Grey-Level Co-occurrence Matrix (GLCM) and Grey-Level Run Length Matrix (GLRLM), as well as histogram-based features. The extracted features were validated against ground-truth data using Spearman’s correlation coefficient (SCC), demonstrating moderate to strong correlations. These findings confirm the robustness of the RW-ensemble segmentation and reinforce the potential of radiomics in enhancing diagnostic accuracy and guiding therapeutic decisions in precision oncology. Establishing the reliability and reproducibility of these features is crucial for their seamless clinical integration, ultimately advancing the role of radiomics in the diagnosis and treatment of lung adenocarcinoma.

## 1. Introduction

**Figure 1:**
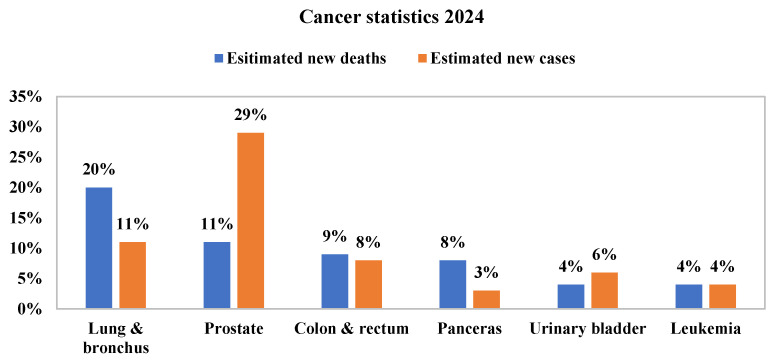
Cancer statistics 2024: Estimated new deaths and estimated new cases [2].

Lung cancer is the leading cause of cancer-related mortality globally, as reported in the 2024 International Agency for Research on Cancer study [1]. According to the World Health Organisation, it accounts for nearly 1.7 million deaths annually, surpassing other cancers such as liver, stomach, colorectal, and pancreatic cancers. Moreover, the global mortality rate is projected to rise significantly, reaching around 17 million by 2030. The American Cancer Society also highlights that lung cancer leads to the highest number of estimated cancer-related deaths in men, as shown in Figure 1 [2].

The primary objective of lung cancer therapy is to deliver effective, individualized treatment based on disease-specific characteristics. Precise radiographic evaluation is critical for planning treatment and guiding clinical decisions. Typically, lung cancer manifests as a tumor within or adjacent to the pleural wall, often with irregular growth and the potential for progressive expansion. Despite advances in treatment, including radiotherapy, surgery, and chemotherapy, the 5-year survival rate remains low [3], emphasizing the urgent need for patient-specific therapeutic strategies.

Lung nodules exhibit considerable biological complexity, encompassing both phenotypic and genetic diversity within and across individual nodules. Even nodules of the same histological type may present diverse imaging characteristics. As a result, analyzing these characteristics and understanding inter- and intra-tumor heterogeneity is essential for precision medicine [4]. Robust biomarkers are required to interpret these variations better and support individualized diagnosis and treatment planning.

Radiomics has recently gained significant traction in the analysis of lung CT imaging [5,6]. It enables the extraction of high-dimensional quantitative features from modalities such as CT, MRI, or PET, offering insight into tumor shape, texture, heterogeneity, and function. These features have been utilized in various clinical applications, including tumor characterization, evaluation of treatment response, prognosis prediction, and early disease detection [7]. For instance, radiomic features have been utilized to assess the response to chemotherapy, enabling clinicians to tailor personalized treatment strategies.

However, several challenges hinder the clinical adoption of radiomics. These include the need for standardization of imaging protocols, variability in acquisition across institutions, and issues related to reproducibility. Furthermore, rigorous validation studies are crucial for establishing the robustness of radiomic biomarkers before they can be reliably integrated into clinical workflows. Addressing these limitations is crucial for realizing the full potential of radiomics in precision medicine.

This study proposes an RW-ensemble method for segmenting and extracting radiomic features of lung nodules. By correlating these features with ground-truth data using Spearman’s correlation coefficient, we aim to assess their significance and reliability as predictive biomarkers in lung adenocarcinoma. The goal is to enhance diagnostic precision, inform therapeutic decisions, and contribute to personalized treatment and survival prediction in clinical oncology.

Extensive research has explored the detection and classification of lung tumors using various segmentation approaches. Simultaneously, numerous studies have focused on deriving quantitative imaging features from segmented CT data [8]. These features include spatial, transform-based, edge, boundary, color, shape, and texture characteristics, all of which show promise for predicting survival outcomes in lung cancer patients [9,10,11]. Nevertheless, many prior studies emphasize either segmentation or feature extraction in isolation, often overlooking their integration, which is crucial for optimizing individualized therapy planning.

Segmentation plays a critical role in radiomics by isolating regions of interest, such as nodules, for feature extraction. Early methods, like region-growing techniques [12], extracted 2D and 3d features for survival analysis using Kaplan–Meier plots but struggled with anatomically complex nodules such as juxta-pleural types, leading to inaccuracies. Advanced approaches, such as single-click ensemble segmentation [13], as applied by Grove et al. [14], generated quantitative descriptors, including convexity and entropy. Shayesteh et al. [15] employed the grow cut segmentation strategy [16] to extract 40 radiomic characteristics from 59 patients using the LCT-D dataset. Despite improvements in feature extraction, these methods faced challenges with initialization parameters and variability in outcomes. The advent of deep learning has enhanced segmentation capabilities, as demonstrated by Paul et al. [17], who utilized neural networks for survival prediction. However, they required large datasets and struggled with complex nodules when combined with traditional methods. Tamponi et al. [18] validated 94 features extracted from manually segmented CT images using a 3d slicer and Gini’s coefficient; however, the reliance on manual segmentation and the lack of robust automation remain persistent challenges.

On the other hand, radiomic feature extraction transforms medical imaging data into meaningful quantitative biomarkers [19,20]. Initially proposed in 2012, radiomics has since undergone significant expansion, focusing on texture features within nodules to aid clinicians in their analyses. This extraction primarily serves prognostic and classification purposes, where classification identifies malignancies and lung illnesses, and prognostication evaluates treatment response and survival. By unveiling hidden data layers in conventional CT scans, radiomics provides substantial therapeutic utility. Its most promising applications lie in oncology [21,22] but extend to other diseases. Studies employing radiomics have consistently demonstrated superior tumor characterization, improved prognosis evaluation, and enhanced drug resistance prediction [23,24]. Parmar et al. [25] advanced this work by developing a region-growing volumetric segmentation technique to extract texture, intensity, and shape features from 20 patient datasets, validated against manual delineation by radiologists with intra-class correlation coefficients (ICC). Aerts et al. [26] further revolutionized the field by extracting 440 features from CT images of 422 patients, utilizing the LUNG1 dataset for training, and validating the results across three independent datasets. Leijenaar et al. [27] corroborated the significance of texture-related features by extracting 44 PET/CT-derived features from 35 patients and validating the findings through intraclass correlation coefficient (ICC) scores.

Carvalho et al. [28] introduced delta-radiomics to quantify variations in radiomic features before and after treatment using PET/CT data from 32 and 26 patients, extracting 118 features, including size and texture descriptors. Chaddad et al. [29] demonstrated that radiomic features enhance survival outcome predictions beyond demographic variables by analyzing 24 characteristics from the NSCLC-Radiomics dataset using Kaplan–Meier plots. Sun et al. [30] tested eight machine learning techniques on 283 patients, identifying the Cox proportional hazards model as the optimal approach for survival prediction using 42 extracted features. Yang et al. [31] combined 2D and 3D CT-derived features in a radiomic nomogram, achieving a concordance index of 0.742 for survival prediction, while Huang et al. [32] utilized 203 features to predict survival outcomes in ALK-positive NSCLC patients with a LASSO-based model. Later, Ninamiya and Arimura [33] extracted 216 wavelet-based radiomic features from 205 fused PET and CT images using custom segmented methodologies. Recent studies, such as those by Selvam et al. [34], have identified 10 key radiomic features that can classify or distinguish between malignant and benign lung nodules. Here, a manual segmentation was employed by three radiologists using the ITK-Snap tool. Later, Selvam et al. [35] extended their work to identify COVID-19 nodules from counterparts of other lung nodules, specifically benign and malignant ones. This work has identified three main key radiomic features for the identification of COVID-19, such as skewness, inverse moment normalization, and long-run low grey level emphasis. Lin et al. [36] expanded the scope of radiomics by classifying histological types and cancer staging in NSCLC, extracting 107 features from 309 patients. Wu et al. [37] addressed the challenge of high dimensionality by selecting nine key radiomic features using LASSO from a dataset of 200 patients with small cell lung cancer (SCLC). Similarly, Wang et al. [38] selected 29 features from a set of 1562 features to predict post-operative recurrence risk in NSCLC, integrating clinical data with radiomic features to improve predictive accuracy.

The summary of the above-mentioned related work on radiomic feature extraction is clearly shown in Table 1. The reviewed studies collectively highlight the transformative potential of radiomics in the analysis of lung cancer, particularly in nodule characterization, treatment response evaluation, and survival prediction. However, critical gaps persist, primarily due to reliance on manual segmentation, which introduces variability and limits reproducibility. Additionally, the lack of standardization in imaging protocols and feature extraction methodologies complicates cross-institutional comparisons. While many researchers have focused on extracting and analyzing radiomic features to explore overall survival and nodule classification, these efforts often overlook the fundamental importance of accurate nodule segmentation—a crucial primary step in computer-aided diagnosis (CAD) systems.

Most studies rely on manual delineation or employ basic segmentation techniques, which face significant limitations. Manual segmentation introduces observer variability, while basic techniques may struggle to accurately segment complex nodule types, such as juxta-pleural and juxta-vascular nodules. These challenges can lead to improper radiomic feature extraction, potentially affecting overall survival predictions and individualized therapy planning. Few studies integrate advanced segmentation techniques with robust feature validation, essential for addressing dimensionality reduction and feature redundancy. This review underscores the transformative potential of radiomics in lung cancer analysis, particularly in nodule characterization, treatment response evaluation, and survival prediction. However, challenges in segmentation reliability, feature validation, and standardization persist. This study aims to address these gaps by utilizing the RW-ensemble segmentation method for feature extraction and validation, with a focus on enhancing the accuracy and clinical applicability of radiomics in precision medicine.

## 2. Methodology

**Figure 2:**
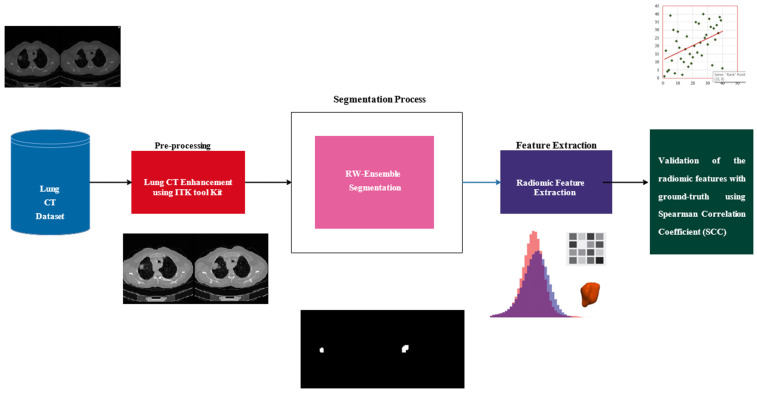
Workflow of the proposed methodology.

This study examines the extraction and validation of radiomic features from lung nodules in CT images, with the goal of establishing their reliability as biomarkers for lung adenocarcinoma. The methodology integrates robust segmentation, feature extraction, and statistical validation techniques. The workflow is illustrated in Figure 2, which begins with a dataset of lung CT images that undergo pre-processing using the ITK Toolkit to enhance image quality through noise reduction and contrast enhancement. Next, the RW-Ensemble segmentation method is applied to delineate regions of interest, such as lung tissues or nodules. Radiomic features, which capture quantitative characteristics such as texture and intensity patterns, are then extracted from the segmented regions. Finally, the extracted features are validated against ground-truth data using the Spearman Correlation Coefficient (SCC) to ensure reliability and consistency. This end-to-end pipeline enables robust and validated radiomic analysis, aiding clinical decision-making and research.

### 2.1. Study Population

**Figure 3:**
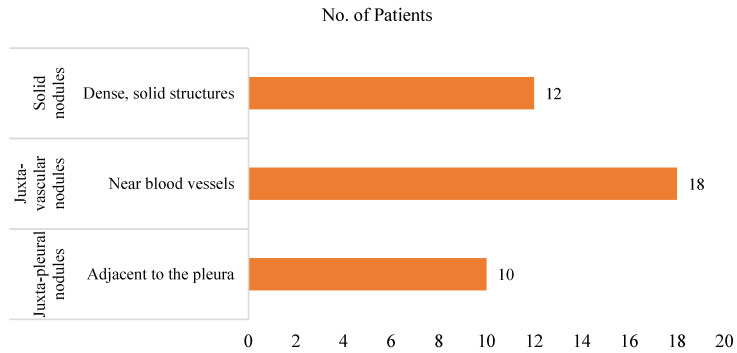
Lung nodule types.

This study leverages the LCT-D dataset from TCIA, comprising data from 61 patients with accompanying clinical information and CT imaging. Segmentation annotations for nodules are available for 40 patients, generated using a radiologist-supervised region-growing technique. These annotations serve as ground truth for validating the study outcomes. The dataset incorporates 37 patients from the LCT-D dataset [39] and three patients from the QIN Lung CT dataset, all diagnosed with lung adenocarcinoma. The nodules are categorized into solid, juxta-vascular, and juxta-pleural. Figure 3 presents a detailed distribution of patients by nodule classification.

All CT slices are formatted as DICOM (.dcm) files. In this work, we utilized the ITK-Snap tool, version 3.6.0-rc1, which is a free-source tool that can read DICOM image formats. We have identified 284 CT images that contain nodules from the dataset. Using this tool, the pre-processing step is also performed, which involves enhancing image contrast with ITK-Snap by selecting “Tools → Image Contrast → Auto-Adjust Contrast” to facilitate improved visualization and diagnostic assessment of the nodules. The use of the ITK-Snap tool ensured robust and standardized handling of image data in the workflow.

### 2.2. RW-E Nodule Segmentation

**Figure 4:**
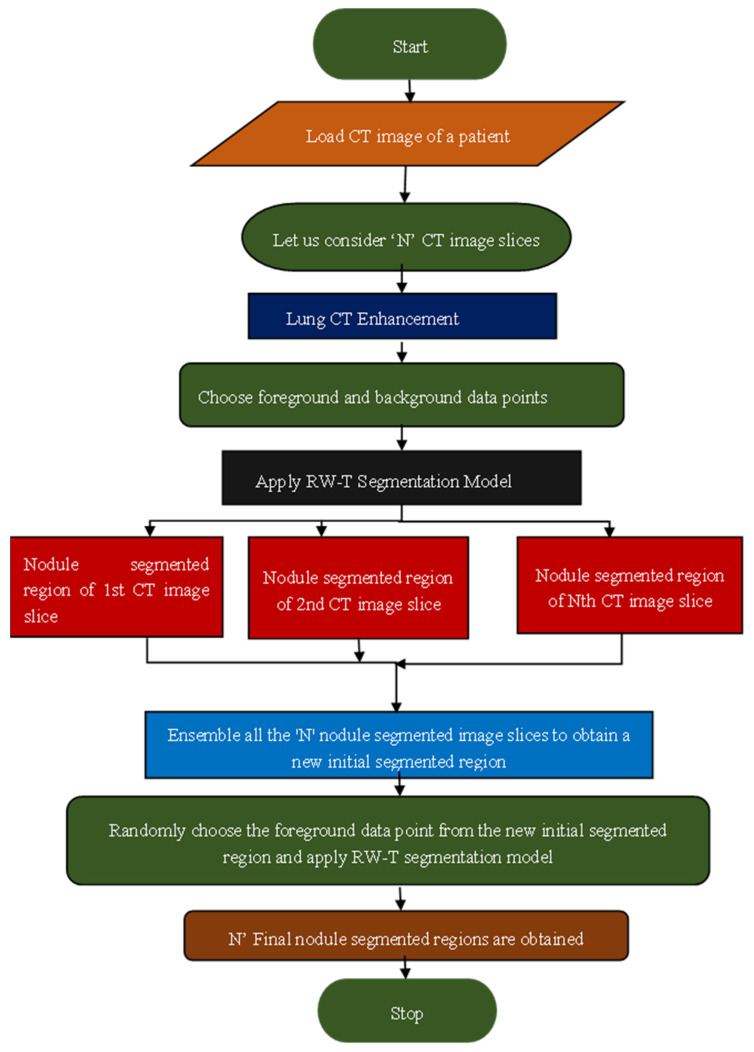
Flowchart representation of RW-E segmentation.

A novel robust segmentation technique, termed RW-E segmentation, was developed to address the limitations of the RW-T hybrid segmentation method. This approach integrates the RW-T segmentation model with an ensemble process to enhance segmentation accuracy and optimize the initialization process. A detailed explanation of the RW-E segmentation process is depicted in the flowchart shown in Figure 4. This methodology ensures accurate and robust segmentation of various lung nodule types, addressing key challenges in medical imaging. The flowchart and algorithm of the RW-E Nodule Segmentation Process are illustrated in Figure 4, which presents a structured approach for segmenting lung nodules from CT image slices.

Here is the step-by-step explanation of the pseudo-code for the RW-E segmentation algorithm, Algorithm 1.

The pseudo-code of the RW-E segmentation algorithm, Algorithm 1.
**Algorithm 1:** RW-Ensemble lung nodule segmentation methodInput: A sequence of lung CT image slices with nodules The execution process:01:    Begin# The dataset is uploaded02: Let the no. of patients be I in the LCT-D dataset 03: **Loop: L1** in the range of I04:      Let the number of CT images be termed as J of Ith patient05:      Consider a variable to store temporary value → temp_val = zeros(size(J)) # **Nodule Segmentation**06:        **Loop: L2** in the range of **J**07:             Select (r1, c1): foreground data point 08:             Select (r2, c2): background data point09:             Implement the RW technique on each CT image.                   J → random_walk_seg_output = random-walk_method (J, (r1,c1), (r2,c2)) 10:             Obtain binary segmented mask using thresholding process 11:             Consider (x, y) = size(random_walk_seg_output)12:             **Loop: L3** in the range (x, y)13:                    Check the condition: random_walk_seg_output (x, y) = th_value 14:                              Allocate → seg_mask1_out (x, y) = 1;15:                    Otherwise16:                              Allocate → seg_mask1_out (x, y) = 0;17:                    Finish18:               **End Loop L3**
# **Ensemble Process**19: Ensemble the temp_val with final segmented output             temp_val = integrate (temp_val, seg_mask1_out)20:        **End Loop L2**21:      Finally, the temp_val gives an ensembled final nodule segmented output 22:      Repeat loop L2 for remaining CT images of a patient I23:      Repeat loop L1 for all remaining patients in the dataset24: **End Loop L1**25: **Stop****Output:** Final segmented region of lung nodule

In the above pseudo code, “I” denotes the number of patients in the LCT-D dataset. Consider a loop “L1” with the range of numbers of patients. The number of CT slices with nodules for each “I^th^“ patient is identified and denoted as “J”. Here, (r1, c1) and (r2, c2) are the foreground and background data points, respectively, chosen to initiate the RW algorithm. Then, the random walk method function applies the RW algorithm to “J” number of CT images and obtains their corresponding grey-scale segmented outputs. These grey segmented images are converted to binary segmented images using the thresholding process illustrated in the loop L3. Later, these “J” number of segmented regions are integrated and termed as an ensemble to obtain a new segmented region. Now repeat the loop L2 for the range of “J” by choosing the foreground and background datapoints from the new segmented region. This ensemble process ensures the proper selection of datapoints, which may be common across all CT slices, and avoids improper segmentation of the lung nodules. In summary, the variables and loops are defined as:

○ I: Denotes the number of patients in the dataset.
○ L1: The loop is iterating over each patient.
○ J: Represents the number of CT slices containing nodules for each patient.
○ L2 and L3: Nested loops for segmentation refinement and thresholding, respectively.
○ (r1, c1) and (r2, c2): Coordinates representing foreground and background seed points selected for initializing the random walk algorithm.

### 2.3. Radiomic Feature Extraction

**Figure 5:**
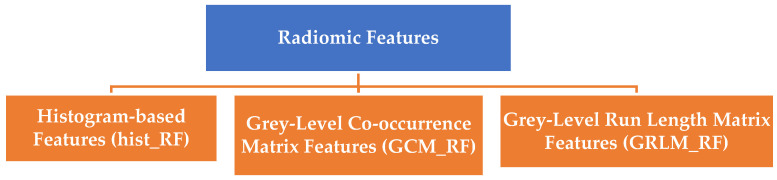
Classification of radiomic features.

Radiomic features play a critical role in extracting quantitative information from medical images, enabling insights into nodule characterization, treatment response evaluation, and survival prediction. These features can be broadly categorized into low-level features, which are directly extracted from the original images, and high-level features, which are derived from the low-level features. The following section outlines these feature classifications, significance, and applications, along with their mathematical formulations and validation techniques. Radiomic features are grouped into three primary categories based on their extraction methodology and clinical implications, illustrated in Figure 5.

Histogram-based Features (hist_RF): Analyze the distribution of pixel intensities in the region of interest (ROI). These features provide first-order statistical measures, such as mean, variance, skewness, and entropy, offering insights into nodule intensity and heterogeneity.

Grey-Level Co-occurrence Matrix Features (GCM_RF): Quantify spatial relationships between pixel intensities to describe texture patterns. Examples include autocorrelation, cluster prominence, and homogeneity.

Grey-Level Run Length Matrix Features (GRLM_RF): Evaluate the occurrence of consecutive pixels with the same intensity, focusing on run lengths. These features capture textural homogeneity and structural patterns, allowing for a more nuanced understanding of the data.

The definitions and notations of radiomic features are represented in Table 2. Columns 1 and 2 represent the definitions and features with their notations. The GCM_RF, GRLM_RF, and hist_RF are explained in detail in the following sub-sections.

Grey-Level Run Length Matrix Features (GRLM_RF): Evaluate the occurrence of consecutive pixels with the same intensity, focusing on run lengths. These features capture textural homogeneity and structural patterns.

#### 2.3.1. Grey-Level Co-Occurrence Matrix Radiomic Feature (GCM_RF)

Grey-Level Co-occurrence Matrix Radiomic Feature is a texture feature. GCM_RF is a statistical measure of the frequency of occurrence of pixels within a specified region. This feature quantifies the texture properties by considering the relationship between the pixels and their neighborhood. It also represents the joint probability distribution of pixel pairs. It characterizes different aspects of texture patterns, enabling the extraction of various texture features. Various texture features associated with GCM_RF are explained below with the aid of formulae.

To define the formulae, let us consider some parameters given below:

N: number of grey levels in an image or any specific region.

$f\left(x,y\right)$ defines the normalized GCM matrix value at position of $\left(x,y\right).$

$\left(x,y\right)$ denotes the row and column position of the GCM matrix.

μ represents the mean of the GCM matrix.

square represents the variance of the GCM matrix.

1. Autocorrelation

It measures similarity among pixels and their neighborhood within their specifically defined region. It quantifies the level of correlation and measures the degree of uniformity among neighborhood pixels. Higher autocorrelation values indicate higher similarity or regularity in texture, while lower values indicate more randomness or heterogeneity. The gcm_rxx given by the Equation (1).

(1) $$gcm\_rxx=\sum_{x=1}^{N}\sum_{y=1}^{N}f(x,y)*{\left(x-\mu \right)}^{2}$$

**Table 2:** Classification of radiomic features.

Radiomic Features	Definition	Features	Denoted As
**GCM_RF**	Determines the spatial correlation between pixels. The texture of an image is determined by analyzing the frequency of pixel pairs with distinct values and specific spatial relationships within the image.	Autocorrelation	gcm_rxx
		Cluster prominence	gcm_cpro
		Cluster shade	gcm_cshd
		Correlation	gcm_corr
		Convexity	gcm_conv
		Contrast	gcm_contr
		Dissimilarity	gcm_disim
		Difference variance	gcm_dvar
		Difference entropy	gcm_dentro
		Energy	gcm_ener
		Entropy	gcm_entro
		Homogeneity	gcm_homo
		Information measure of correlation 1	gcm_imc1
		Information measure of correlation 2	gcm_imc2
		Inverse moment normalized	gcm_imn
		Maximum probability	gcm_max_prob
		Sum average	gcm_savg
		Sum variance	gcm_svar
		Sum entropy	gcm_sentro
**GRLM_RF**	Quantifies the grey level runs by measuring the length, in terms of the number of pixels, of consecutive pixels that have the same grey level value.	Short-run emphasis	grlm_sr
		Long run emphasis	grlm_lr
		Grey-level non-uniformity	grlm_gn
		Run length non-uniformity	grlm_rln
		Run percentage	grlm_rper
		High grey-level run emphasis	grlm_hgr
		Low grey-level run emphasis	grlm_lgr
**hist_RF**	Analyze the range of pixel intensities within the area of interest (ROI) using standard and fundamental metrics.	Energy	hist_ener
		Entropy	hist_entro
		Skewness	hist_skew
		Kurtosis	hist_kurt
		Maximum	hist_max
		Minimum	hist_min
		Mean	hist_mean
		Median	hist_median
		Mean absolute difference	hist_mad
		Standard deviation	hist_sd
		Range	hist_range
		Variance	hist_var

2. Cluster prominence

Cluster prominence quantifies the degree of asymmetry in the GCM_RF, with higher values indicating more significant skewness in the distribution of intensity pairs. It provides information about the arrangement of the co-occurring intensity values. A positive value of gcm_cpro indicates a predominance of intensity pairs, while a negative value gives a more balanced distribution of pixels. Equation (2) is as follows:

(2) $$gcm\_cpro=\sum_{x=1}^{N}\sum_{y=1}^{N}f(x,y)*{\left(x+y-2\mu \right)}^{4}$$

3. Cluster shade

The cluster shade quantifies the asymmetry in the GLCM distribution, with higher values indicating more significant skewness. It provides information about the arrangement of co-occurring grey-level values in the image texture. The formula to compute cluster shade is referred to as Equation (3).

(3) $$gcm\_csh=\sum_{x=1}^{N}\sum_{y=1}^{N}f(x,y)*{\left(x+y-2\mu \right)}^{3}$$

4. Convexity

Convexity is a texture feature that measures the degree of convexity or concavity of the GCM_RF distribution. In radiomics, convexity represents a crucial aspect of shape-based analysis, providing insights into the geometric characteristics of structures observed in images and their potential clinical significance. The changes in convexity over time or in response to treatment may provide valuable information for monitoring disease progression or evaluating treatment efficacy. The formula for convexity is shown in Equations (4) and (5).

(4) $$Convexity=\frac{area(perimeter(nodule))}{area(convexhull(nodule))}$$

(5) $$Total\_convexity=mean_{\left(convexity\;of\;all\;CT\;slices\right)}$$

A high convexity value indicates a smoother and more regular shape, while a lower convexity value suggests irregularities or concavities within the structure.

5. Dissimilarity

Dissimilarity measures the average absolute difference in intensity values among neighboring pixels. It quantifies the heterogeneity or variation in the texture. Greater dissimilarity values indicate increased variance in texture, whereas lower values reflect more uniformity or homogeneity. It is defined by the formula shown in Equation (6).

(6) $$gcm\_disim=\sum_{x=1}^{N}\sum_{y=1}^{N}f(x,y)*(\left|x-y\right|)$$

6. Energy

Energy is computed by summing the squared elements of the GCM matrix. Squaring, followed by summing, indicates the overall homogeneity or uniformity of the texture in a nodule. Greater energy values signify a more consistent texture, whereas lower values indicate more variability. Equation (7) defines gcm_ener as follows:

(7) $$gcm\_ener=\sum_{x=1}^{N}\sum_{y=1}^{N}f{\left(x,y\right)}^{2}$$

7. Entropy

Entropy measures the randomness or uncertainty of an image’s texture and quantifies the amount of information or disorder present in the texture. Lower values suggest more regularity or homogeneity, whereas higher entropy values indicate more randomness or heterogeneity in the texture. It is calculated using the formula in Equation (8).

(8) $$gcm\_entro=\sum_{x=1}^{N}\sum_{y=1}^{N}f(x,y)*\log\;f(x,y)$$

8. Difference entropy

Difference entropy measures the randomness or uncertainty of the differences between pixel intensity values in an image. It quantifies the randomness in the distribution of the differences between neighboring pixel intensities. Higher values of gcm_dentro indicate greater variability or randomness in these differences, while lower values suggest greater uniformity or predictability. Equation (9) defines the formula for computing gcm_dentro.

(9) $$gcm\_dentro=\sum_{k=1}^{2N-1}f_{diff}(k)*\log\;f_{diff}(k)$$

Here, denotes the normalized GCM value for the difference between neighboring pixel intensities.

9. Sum entropy

Sum entropy is similar to GCM entropy; instead of the difference, a sum of pixel intensities is considered for its calculation. It measures the randomness or uncertainty of the sum of pixel intensity values in an image. The gcm_sentro quantifies the randomness in the distribution of the sums of neighboring pixel intensities. Higher values of gcm_sentro indicate more significant variability or randomness in these sums, while lower values suggest more uniformity or predictability. It is represented as Equation (10).

(10) $$gcm\_sentro=\sum_{k=1}^{2N-1}f_{sum}(k)*\log\;f_{sum}(k)$$

Here, denotes the normalized GCM value for the sum between neighboring pixel intensities.

10. Homogeneity

Homogeneity quantifies the similarity or closeness of neighboring pixel pairs in terms of their grey-level values. Higher homogeneity values indicate more uniformity or regularity in the texture, whereas neighboring pixels have similar intensities. Lower values indicate greater variability or heterogeneity in the texture. The formula is defined as follows using Equation (11).

(11) $$gcm\_homo=\sum_{x=1}^{N}\sum_{y=1}^{N}\frac{f(x,y)}{1+\left|x-y\right|}$$

11. Information measure of correlation 1

Information measure of correlation 1 measures the linear dependency between the grey-level values of neighboring pixels. It quantifies the correlation between the grey-level values of neighboring pixels in a region. The more excellent value of gcm_imc1 indicates a linear relationship among pixel intensities, while the lower value represents a non-linear relationship. Equation (12) gives the formula to calculate gcm_imc1.

(12) $$gcm\_imc1=\frac{\sum_{x=1}^{N}\sum_{y=1}^{N}f(x,y)*(x-\mu )*(y-\mu )}{\sigma^{2}}$$

12. Information measure of correlation 2

Information measure of correlation 2 is similar to gcm_imc1. It measures the linear dependency between neighboring pixels’ grey-level values and the normalized version of gcm_imc1 which is shown in the Equation (13).

(13) $$gcm\_imc2=\frac{\sum_{x=1}^{N}\sum_{y=1}^{N}f(x,y)*\frac{\left(x-\mu )*(y-\mu \right)}{\sigma^{2}+1}}{\sigma^{2}}$$

13. Inverse moment normalized

Inverse moment normalized is a measure of similarity or homogeneity of the texture in an image. Higher values indicate more uniformity or homogeneity in the texture, whereas neighboring pixels have similar intensities. Lower values indicate greater variability or heterogeneity in the texture. Equation (14) represents it as follows.

(14) $$gcm\_imn=\sum_{x=1}^{N}\sum_{y=1}^{N}\frac{f(x,y)}{1+{\left(x-y\right)}^{2}}$$

14. Maximum probability

Maximum probability represents the probability of the most frequently occurring pair of pixel values in a specified region. It is calculated as follows using Equation (15).

(15) gcm_max_prob=max(f(x,y))

15. Sum average

The sum average represents the average sum of the grey-level pairs in the GLCM matrix, weighted by their distances from the main diagonal. Higher values of the gcm_savg indicate a greater concentration of grey-level pairs around the main diagonal, whereas lower values suggest a more spread-out distribution. Equation (16) is used to calculate gcm_savg.

(16) $$gcm\_savg=\sum_{k=1}^{2N-1}f_{sum}(k)*k$$

$f_{sum}\left(k\right)$ is the normalized value representing the probability of occurrence of a given the sum k n the GCM.

16. Sum variance

Sum variance is calculated with the help sum average and measures the variance of the sum of intensity values in the GCM matrix. It is denoted as gcm_svar, and the formula is defined by Equation (17).

(17) $$gcm\_svar=\sum_{k=1}^{2N-1}f_{sum}(k)*(k-gcm\_savg)$$

17. Difference average

The difference average is similar to the gcm_savg. It measures the average difference of the grey-level pairs in the GLCM matrix, weighted by their distances from the main diagonal instead of the average sum of intensity pairs. It is calculated using a formula defined by Equation (18).

(18) $$gcm\_davg=\sum_{k=1}^{2N-1}f_{diff}(k)*k$$

18. Difference variance

Difference variance is similar to gcm_svar and measures the variance of the difference of intensity values in the GCM matrix instead of the sum variance. It is calculated using the formula given by Equation (19).

(19) $$gcm\_dvar=\sum_{k=1}^{2N-1}f_{diff}(k)*(k-gcm\_svar)$$

#### 2.3.2. Grey-Level Run Length Matrix Radiomic Feature (GRLM_RF)

The grey-level run length matrix radiomic feature is denoted as GRLM_RF. The GRLM_RF matrix represents each element as a specific intensity and run length pair. Various statistical features were extracted from the GRLM matrix, and it also performs texture analysis to quantify and characterize the spatial distribution of homogeneous patterns within an image. In this feature analysis, a “run” refers to a sequence of consecutive pixels within an image that shares the same grey-level value and is connected horizontally, vertically, or diagonally.

When constructing a GRLM_RF matrix, the image is first quantized into a certain number of grey levels, typically ranging from 8 to 256. Then, the matrix is generated by counting the number of consecutive pixel runs for each grey level and run length combination in different directions. Each element of the GRLM_RF represents the frequency of occurrence of a specific grey-level run length pair within the image. These frequency values are computed for different directions and different grey levels, providing information about the texture and spatial patterns present in the image. Overall, runs in GRLM_RF analysis help characterize the texture of an image by quantifying the distribution of homogeneous patterns of different lengths and grey levels.

The various types of features that come under GRLM_RF are demonstrated below. To define the formulae, let us consider some parameters given below:

N: number of grey levels in an image or any specific region.

$P\left(x,y\right)$ denotes the probability of occurrence of runs of length y with intensity level x.

$N_{r}$ is the maximum run length, where y length of the run.

1. Short-run emphasis

The short-run emphasis provides valuable insights into the spatial distribution of short homogeneous runs within an image, aiding in texture analysis. It quantifies the emphasis on short, consecutive runs of homogeneous grey-level values within a region or an image. The grlm_sr emphasizes the significance of short, continuous areas in the image when the grey-level values are consistent. It emphasizes capturing complex details and rapid variations in intensity levels in the image. It is calculated using the formula given by Equation (20).

(20) $$grlm\_sr=\frac{\sum_{x=1}^{N}\frac{p(x,y)}{y^{2}}}{\sum_{x=1}^{N}\sum_{y=1}^{N}\frac{p(x,y)}{y^{2}}}$$

2. Long run emphasis (grlm_lr)

The long-run emphasis in GLRLM measures the joint occurrence of long runs with high grey-level values in an image. It quantifies the tendency of longer runs of high-intensity values to occur, which can provide insights into the texture characteristics of the image. A higher long-run emphasis value indicates that longer runs of high-intensity values are more prominent in the image, potentially indicating specific textural patterns or structures. It is calculated using the formula given in Equation (21).

(21) $$grlm\_lr=\sum_{x=1}^{N}\sum_{y=1}^{N}\frac{p(x,y)}{y^{2}}$$

3. Grey-level non-uniformity

Grey level non-uniformity measures the variability of grey levels along the runs in a specified region or an image. A more excellent value represents higher variability in grey-level values, indicating a more heterogeneous texture. In contrast, a lower value represents a more homogeneous texture with less variability in intensity values. It can be defined using Equation (22).

(22) $$grlm\_gn=\sum_{x=1}^{N}\frac{\sum_{y=1}^{N_{r}}p{\left(x,y\right)}^{2}}{N_{r}}$$

Here $N_{r}(r)$ represents the number of runs in a specific run length r.

4. Run length non-uniformity

Run length non-uniformity measures the variability in run length in a region or an image. A lower value represents a more regular texture in terms of length of intensity values, while a higher value defines a more irregular texture. It is given by Equation (23).

(23) $$grlm\_rln=\sum_{x=1}^{N_{r}}\frac{\sum_{y=1}^{N}p{\left(x,y\right)}^{2}}{N}$$

5. Run percentage

Run percentage gives insight into the distribution of run lengths within the image. It indicates the relative frequency of runs of a specific length compared to the total number of runs. High values of grlm_rper for a particular run length indicate that runs of that length are prominent in the image, while low values indicate that runs of that length are less common. Equation (24) is represented as follows, where $N_{r}(r)$ represents the number of runs in a specific run length r.

(24) $$grlm\_rper=\frac{N_{r}(r)}{N}*100\%$$

6. Low grey-level run emphasis (grlm_lgr)

Low grey-level run emphasis measures the tendency of longer runs with low grey-level values to occur. A higher grlm_lgr value indicates that longer low-intensity values are more prominent in the image, suggesting specific textural patterns or structures characterized by low-intensity levels. The formula is defined by using Equation (25).

(25) $$grlm\_lgr=\sum_{x=1}^{N}\sum_{y=1}^{N}\frac{p(x,y)}{x^{2}}$$

7. High grey-level run emphasis (grlm_hgr)

High grey-level run emphasis measures the tendency of longer runs with high grey-level values to occur. A higher value of grlm_hgr indicates that longer runs of high-intensity values are more prominent in the image, suggesting specific textural patterns or structures characterized by high-intensity levels. Equation (26) gives the formula to calculate this feature.

(26) $$grlm\_hgr=\sum_{x=1}^{N}\sum_{y=1}^{N}\frac{p(x,y)*x^{2}}{y^{2}}$$

8. Histogram-based features

Histogram-based radiomic features are derived from the histogram of intensity values in CT images. These features aim to measure the statistical information about the distribution of pixel intensities and can be used for analyzing the nodule characterization, treatment response assessment, and patient outcome prediction. Here, histogram-based radiomic features and their formulas are given as follows. Here, let us consider the following:

N: total number of pixels.

$f_{i}$: denotes the intensity value of the ith pixel.

9. Mean (hist_mean)

The mean is calculated by considering the mean of the histogram and is given by the formula using Equation (27).

(27) $$hist\_mean=\frac{1}{N}\sum_{i=1}^{N}f_{i}$$

10. Median

The median is calculated by considering a histogram’s median of intensity values.

11. Maximum

The maximum intensity value of the histogram is represented as the maximum value.

12. Minimum

The minimum intensity value of the histogram is represented as the minimum value.

13. Standard deviation

Standard deviation measures the dispersion of intensity values around the mean and is given by Equation (28).

(28) $$hist\_sd=\sqrt{\frac{1}{N}\sum_{i=1}^{N}\left(f_{i}-hist\_mean\right)}$$

14. Range

The difference between the maximum and minimum values obtained from the histogram is a range.

15. Mean absolute difference

It quantifies the average absolute difference between individual data points and the mean of the data.

16. Energy

Energy is calculated by taking the sum of squares of all histogram values. The formula is given below using Equation (29).

(29) $$hist\_ener=\sum_{i=1}^{N}(f_{i}{)}^{2}$$

17. Entropy (hist_entro)

Entropy is the measure of randomness in the histogram distribution. It is given below using Equation (30).

(30) $$hist\_entro=-p_{i}*log\;p_{i}$$

18. Skewness

Skewedness is the measure of asymmetry in the histogram distribution. The formula is given using Equation (31).

(31) $$hist\_skew=\frac{\frac{1}{N}\sum_{i=1}^{N}{\left(f_{i}-hist\_mean\right)}^{3}}{hist\_sd^{3}}$$

19. Kurtosis

Kurtosis measures the histogram distribution’s peak value and is defined using the following Equation (32).

(32) $$hist\_kurt=\frac{\frac{1}{N}\sum_{i=1}^{N}{\left(f_{i}-hist\_mean\right)}^{4}}{hist\_sd^{4}}$$

Here, Table 3 provides an organized summary of the radiomic features extracted for texture, shape, and intensity analysis. It categorizes the features into groups based on their type and function, including texture features, which describe variations and uniformity in pixel intensities; cluster-based features, which quantify skewness and asymmetry in intensity distribution; and shape features, focusing on geometric regularity. Additionally, it includes information correlation metrics, which measure the dependency between pixel intensities, and sum/difference metrics, which assess variations in sums or differences of pixel intensities. The Grey-Level Run Length Matrix (GLRLM) features characterize the distribution of continuous intensity runs. In contrast, histogram-based features summarize intensity distributions statistically, providing critical insights into the texture and structural properties of the image.

The radiomic features analyzed in this study provide a comprehensive characterization of image textures, shapes, and intensity distributions, offering valuable insights into the underlying patterns and structures. Texture features such as autocorrelation, cluster-based metrics (prominence, shade, and contrast), homogeneity, dissimilarity, and entropy capture local and global variations, while convexity and information measures of correlation describe geometric and dependency relationships among pixel intensities. Advanced metrics derived from the Grey-Level Co-occurrence Matrix (GLCM) and Grey-Level Run Length Matrix (GLRLM), including short and long-run emphasis, non-uniformity, and run percentage, further detail spatial and intensity-related distributions. Histogram-based features like mean, standard deviation, skewness, and kurtosis provide statistical summaries of intensity distributions, complementing the analysis. These features collectively enable robust quantitative evaluation of image characteristics, forming the foundation for the subsequent results and interpretations presented in this section. This tabular representation condenses the detailed descriptions into a concise format for more straightforward interpretation and reference in the Section 3.

## 3. Result Analysis

**Figure 6:**
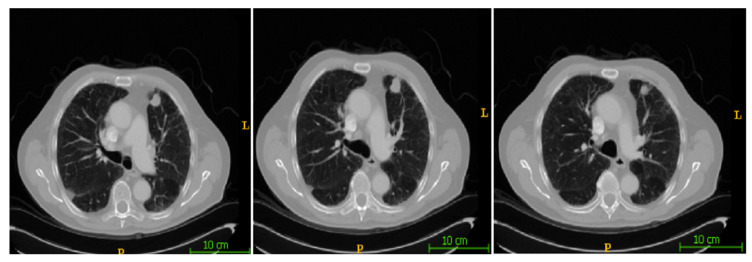
CT slices showing a solid lung nodule (Patient R_0108).

**Figure 7:**
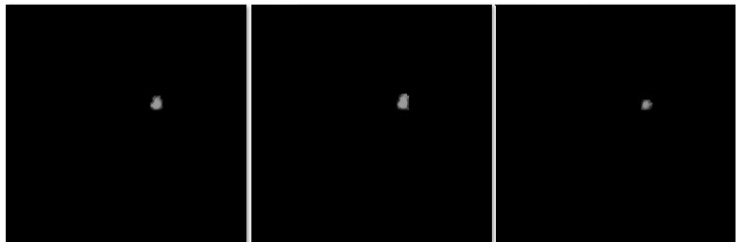
RW-ensemble segmentation output of the solid nodule in grey-scale.

**Figure 8:**
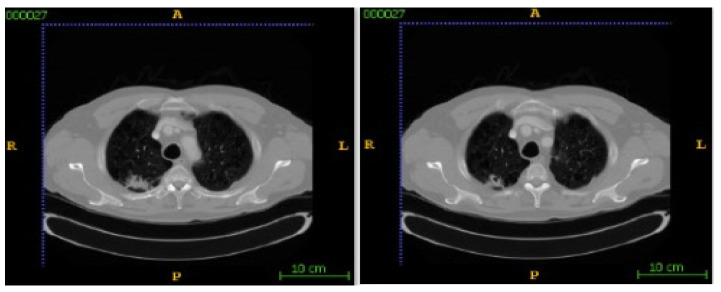
CT slices showing juxta-vascular nodules (Patient R_0052).

**Figure 9:**
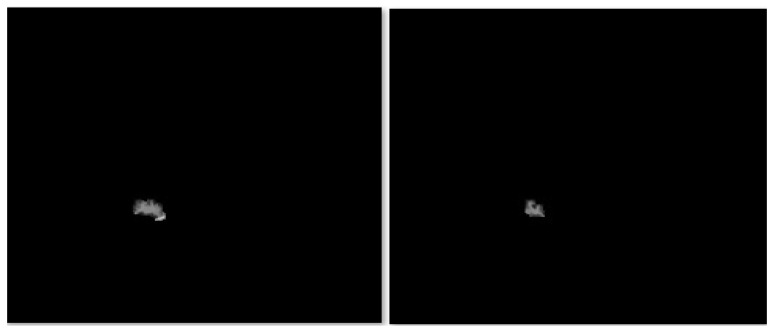
RW-ensemble segmentation output of juxta-vascular nodules in grey-scale.

**Figure 10:**
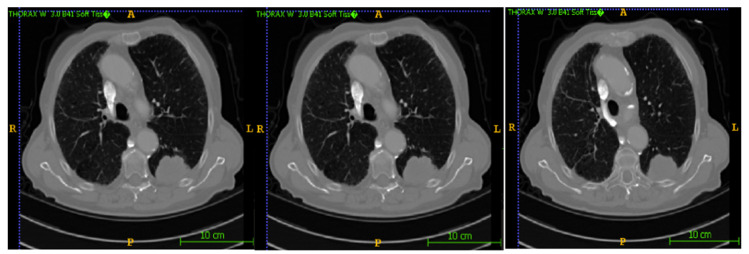
CT slices showing juxta-pleural nodules (Patient QIN_LSC_0064).

**Figure 11:**
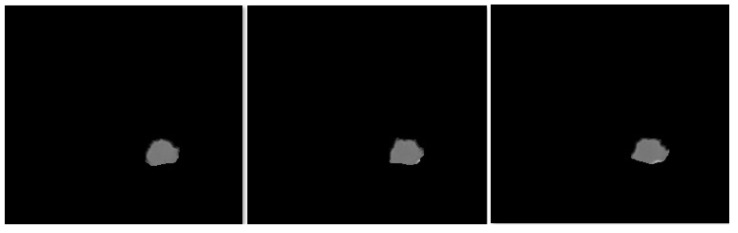
RW-ensemble segmentation output of juxta-pleural nodules in grey-scale.

In this section, the segmentation results of the RW-ensemble are visualized for distinct types of nodules. The results for three different types of nodules from the LCT-D dataset are demonstrated: solid nodules, juxta-vascular, and pleural nodules. To improve clarity, each case is visualized through CT slices and the corresponding RW-ensemble segmented outputs, organized by nodule type and patient ID. Figure 6 and Figure 7 represent the solid nodule and its respective RW-E segmented images in grey-scale for patient R_0108. The juxta-vascular nodules of patient R_0052 are shown in Figure 8, and the corresponding RW-E segmented output images are illustrated in Figure 9. Juxta-pleural nodules, which are challenging due to their attachment to the lung wall, are illustrated in Figure 10 and Figure 11 for patient QIN_LSC_0064.

Next, the radiomic features were extracted from the segmented nodules that were obtained from the proposed method and from the ground-truth nodules of the Lung-CT dataset. The goal was to assess how closely the features derived from RW-ensemble segmentation match the features obtained from the annotated ground-truth data. Later, the correlation analysis of radiomic features with respect to the ground-truth features is visualized in this section. Here, thirty-eight radiomic features have been derived from the segmented nodule output of the RW-ensemble segmentation approach, including 26 texture features and 12 histogram-based features using MATLAB 2020a. GCM_RF and GRLM_RF are popular methods for extracting texture characteristics from images. A total of 19 GCM_RF and 11 GRLM_RF were calculated using the formulas mentioned earlier. These features are calculated using radiomic tools in MATLAB [40]. Each feature was computed by averaging all CT slices containing identified nodules from a specific patient.

The extracted radiomic features were further validated concerning the ground-truth labeled features using SCC. These ground-truth-labeled features were extracted from the labeled segmentation annotations provided by the Lung CT-Diagnosis dataset in TCIA. The results of this validation are presented using scatter plots and correlation coefficient tables to clearly show the strength of association between each pair of features.

Next, a feature selection step is considered, which plays a vital role in reducing the dimensionality of feature space and eliminating feature redundancy by selecting a subset of the radiomic features while retaining the most relevant and informative features. Here, 12 features of GCM_RF, six features of GRLM_RF, and six features of hist_RF are selected, which retain information and avoid redundancy. These selected features were chosen based on their correlation strength and consistency across multiple cases.

Researchers also seek to establish the statistical significance of their findings. A *t*-test can assess whether the correlation coefficient is equal to zero. The *p*-value obtained from the test does not indicate the strength of the relationship between the two variables. Large datasets can yield statistically meaningful results even with very tiny correlation coefficients. Thus, a statistically significant correlation should not be mistaken for a clinically meaningful correlation [41].

Correlation coefficients are statistical measures that quantify the degree of association or relationship between two variables. Therefore, in radiomics, correlation coefficients can be used to assess the relationship between different image features and features obtained from ground-truth segmented images. There are two main types of correlation coefficients: the Pearson correlation coefficient and Spearman’s correlation coefficient. The Pearson correlation coefficient assumes a linear relationship between the features and is suitable for normally distributed data. Spearman’s correlation coefficient does not assume a linear relationship between the features and is a more robust correlation in case of a non-linear relationship between the data. Therefore, in this study, Spearman’s correlation coefficient is used to perform the correlation between the features.

Spearman’s correlation coefficient is denoted as SCC. It is a statistical measure of the strength of the monotonic relationship between pairs. The range of SCC is between −1 and +1. The value “+1” indicates perfect positive monotonic correlation between the paired features, and the value “−1” indicates perfect negative monotonic correlation between paired features. The value “0” indicates no correlation. If the value is in the range of −1 to 0 represents a negative relationship, and 0 to 1 represents the positive relationship between the two features.

Here, the SCC is calculated for three types of radiomic features. These include histogram-based, GCM_RF, and GRLM_RF features. The following procedure calculates the SCC for two radiomic feature pairs. This pair includes the features extracted from the proposed segmentation method, and others are considered from the ground truth. The resulting SCC values help identify which radiomic features are most consistently reproducible, offering insights into their reliability for clinical application.

The procedure for the calculation of SCC is as follows:

- Calculate the ranks of each feature separately.
- Calculate the difference between the ranks of each observation.
- Sum the squared differences in ranks.
- The formula to calculate SCC is given by Equation (33).

(33) $$SCC=1-\frac{6*\sum d_{i}^{2}}{n(n^{2}-1)}$$

where SCC represents Spearman’s correlation coefficient, $d_{i}$: is the difference between the ranks of each pair of observations, and n: is the number of paired observations.The above procedure calculates the SCC values for GCM_RF, GRLM_RF, and hist_RF. A positive monotonic relationship between the pair of values is observed. The strength of SCC is given by Ali Abd Al-Hameed [42] and is represented in Table 4. The negative monotonic relationship is the exact reverse strength of the positive monotonic relationship.

According to the above analysis, a positive monotonic relationship is identified between the GCM_RF feature pairs. Out of 19 GCM_RF features, 12 are considered the most significant and calculated for SCC. The SCC values with their strengths are given in Table 5, where six features represent weakly, and the other six represent moderate monotonic positive relationships between the pair. It has been reported that the convexity feature has a strong positive monotonic relationship with the pairs.

The SCC values of GRLM_RF are calculated and represented in Table 6. The three features of GRLM_RF exhibit a solid positive monotonic relationship. The remaining reported weak and moderate positive relationships are shown in Table 7. The SCC values for hist_RF are given in Table 8. Out of 11 features, the SCC values are calculated for 6 significant hist_RF features. Almost all features exhibited moderate to solid strength, except the hist_kurt feature, which reported a weak monotonic relationship.

**Figure 12:**
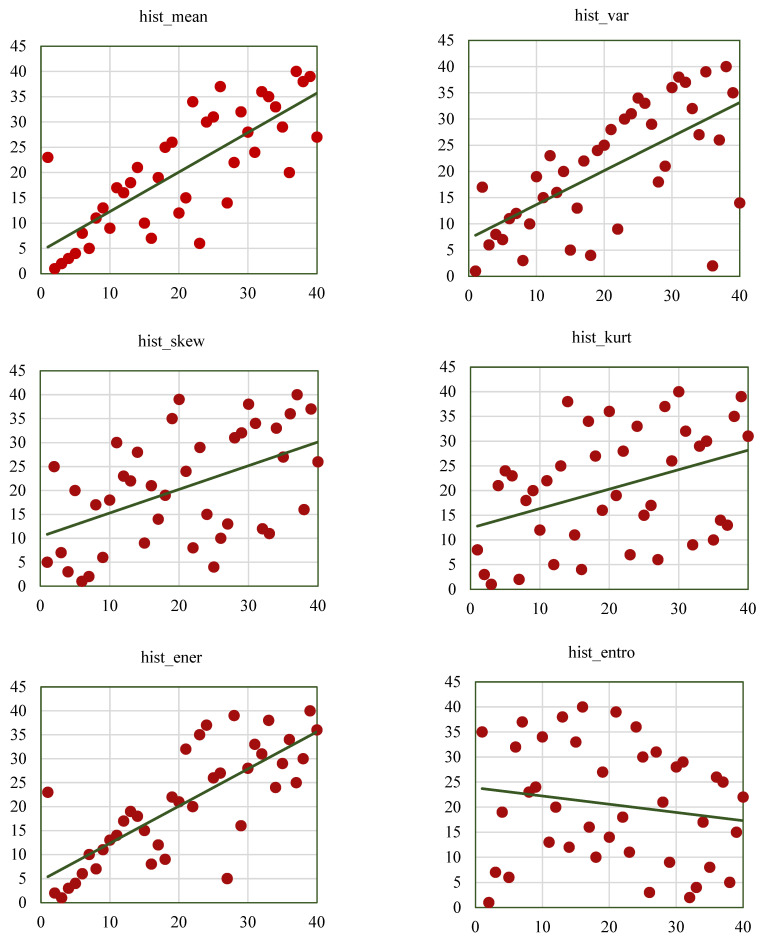
Scatterplots of SCC ranks for histogram-based radiomic features. The green line indicates the ideal line of perfect rank correlation (y = x), serving as a reference for agreement between ranks. The red circle points highlight a key feature of interest with a notable correlation outcome, such as a particularly high or low SCC value.

**Figure 13:**
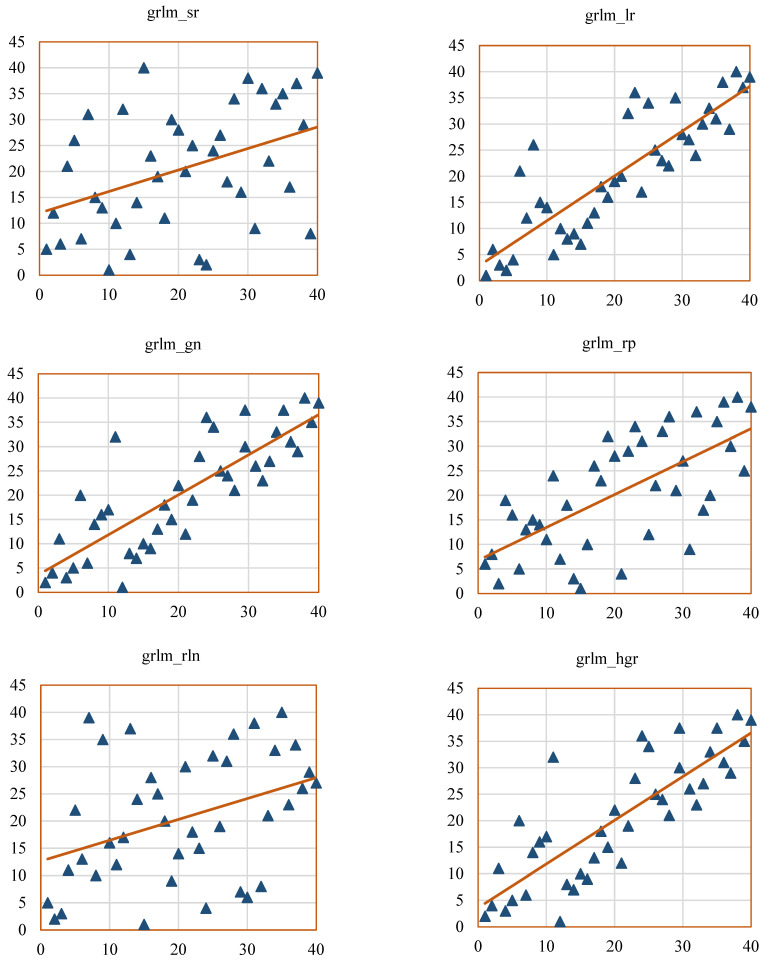
Scatterplots of SCC ranks for grey-level run length matrix (GRLM_RF) features. The orange line represents the ideal line of perfect rank correlation (y = x), while the blue triangle highlights a key feature of interest with a notably high or low correlation score.

**Figure 14:**
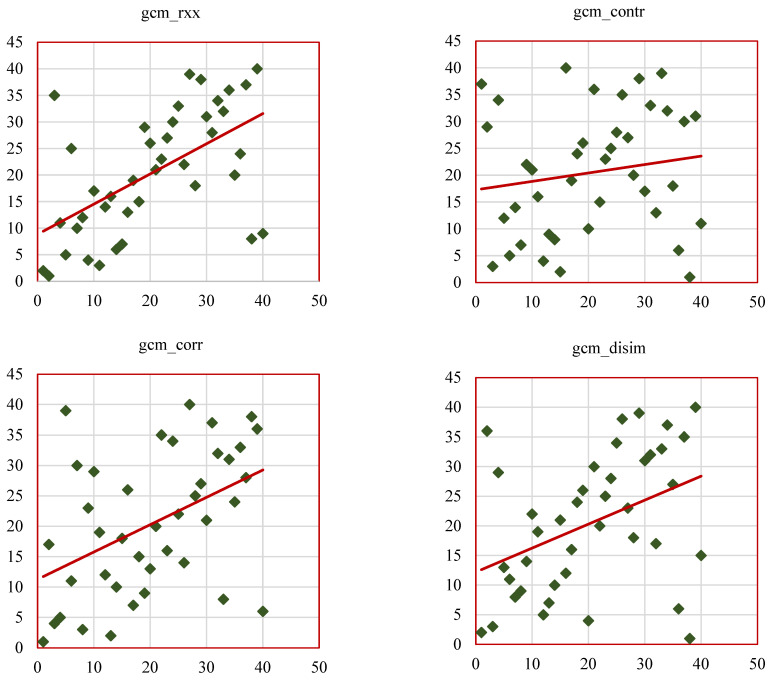

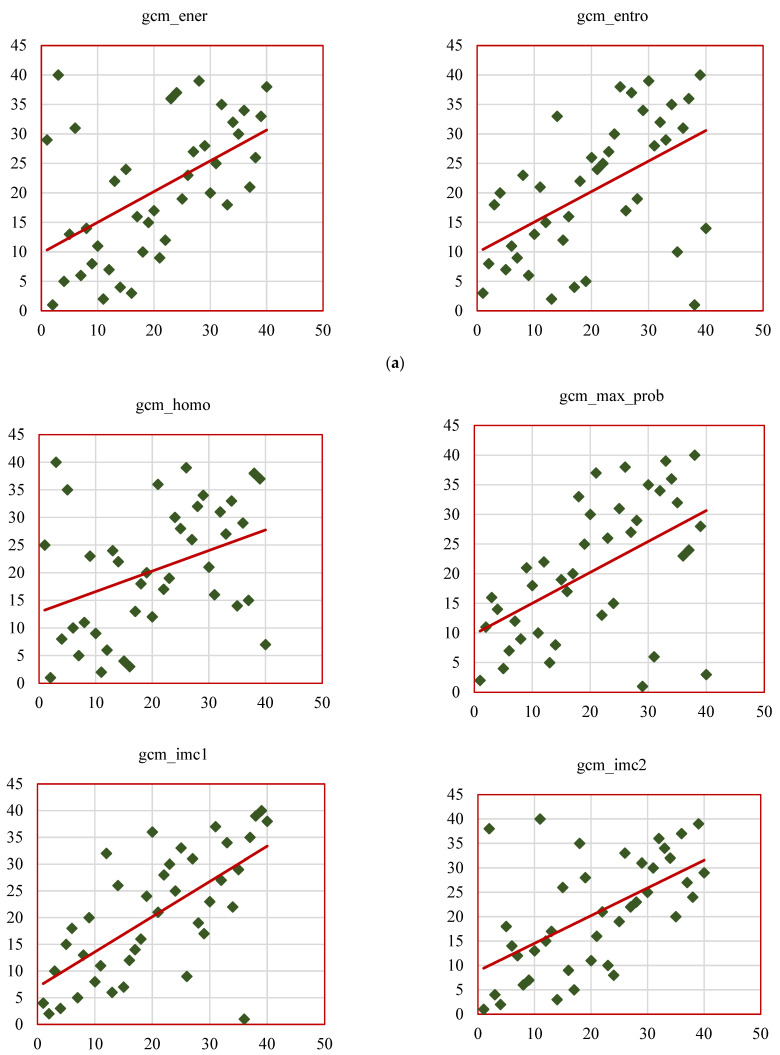

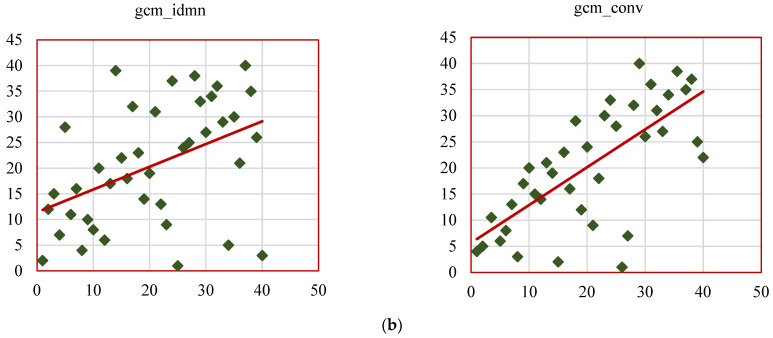
(**a**) Scatterplots of SCC ranks for selected GCM_RF features (Set 1). (**b**) Scatterplots of SCC ranks for selected GCM_RF features (Set 2). The green squares and red lines represent the ideal line of perfect rank correlation (y = x), and a key feature of interest with a notably high or low correlation score respectively.

Scatterplots help to visualize the SCC values of the radiomic features, such as GCM_RF, GRLM_RF, and hist_RF. Figure 12, Figure 13 and Figure 14 represent the scatterplots of the SCC ranks of hist_RF, GLRM_RF, and GCM_RF. For each scatterplot, the *x*-axis represents the ranks of the observations corresponding to the features extracted from the ground-truth segmentation annotations from the Lung CT-Diagnosis dataset, and the *y*-axis represents the ranks of observations related to the features extracted from the RW-ensemble segmentation implemented on the same dataset.

Figure 12 illustrates the scatterplots of the hist_RF features such as hist_mean, hist_var, hist_skew, hist_kurt, hist_ener, and hist_entro. Figure 13 displays the scatterplots of SCC ranks GLRM_RF features such as grlm_sr, grlm_lr, grlm_gn, grlm_rln, grlm_rper, and grlm_hgr.

Figure 14a illustrates the scatterplots of SCC ranks of GCM_RF features such as gcm_rxx, gcm_contr, gcm_corr, gcm_disim, gcm_ener, gcm_entro. Figure 14b represents the scatterplots of SCC ranks of remaining GCM_RF features such as gcm_homo, gcm_max_prob, gcm_imc1, gcm_imc2, gcm_imn, gcm_conv. The results of this study align with existing research, reinforcing the value of radiomic features as biomarkers for lung cancer characterization while introducing novel insights through the RW-ensemble segmentation method. Similar to Ganeshan et al. [9] and Aerts et al. [26], the study highlights the clinical significance of texture features. High grey-level run emphasis (GRLM_RF, SCC = 0.82) and skewness (histogram, SCC = 0.83) align with Aerts et al.’s findings on the predictive power of texture-based radiomics for survival analysis. Shape features, particularly convexity (GCM_RF, SCC = 0.73), reaffirm their importance as indicators of tumor geometry and progression, consistent with Huang et al. [32].

While earlier works relied heavily on manual segmentation or single-modality approaches, this study’s RW-ensemble segmentation improves delineation accuracy for complex nodules, such as juxta-pleural and juxta-vascular types, thereby reducing observer variability. In most of the studies that rely heavily on manual segmentation or neural network-based approaches (e.g., Tamponi et al. [18]), this paper employs the RW-ensemble segmentation method, which enhances robustness and demonstrates higher consistency in feature validation. Unlike studies such as Parmar et al. [25] that focus on linear relationships (e.g., intra-class correlation coefficients), the use of Spearman’s correlation coefficient (SCC) provides robustness in capturing monotonic relationships, but this methodology could miss non-linear interactions present in real-world data.

Several features displayed weak correlations, such as kurtosis (histogram-based, SCC = 0.37) and dissimilarity (GCM_RF, SCC = 0.38), which can be attributed to several factors. Feature redundancy, where metrics like kurtosis overlap with other statistical measures, may dilute their independent contribution to tumor characterization.

Additionally, dataset limitations, despite their robustness, might lack sufficient diversity or variability in imaging protocols and nodule types, preventing certain features from fully showcasing their potential. Clinically, while these weakly correlated features may have limited standalone utility, they could still provide value as complementary metrics in multivariate analyses, contributing to broader insights into tumor behavior.

The strongly correlated features identified in this study have significant potential for clinical application. Features like skewness (histogram-based, SCC = 0.83) and high grey-level run emphasis (GRLM_RF, SCC = 0.82) could assist in the early differentiation of malignant versus benign nodules, potentially reducing the need for invasive diagnostic procedures. Additionally, convexity (GCM_RF, SCC = 0.73) offers valuable insights into tumor geometry, helping predict tumor invasiveness and guiding radiotherapy or surgical treatment plans. The comparative analysis of the above studies is presented in Table 8.

Validated radiomic features from this study hold significant potential for enhancing clinical workflows. Features such as skewness (histogram) and convexity (GCM_RF) can improve early diagnosis by enabling differentiation between malignant and benign nodules at an earlier stage. High grey-level run emphasis (GRLM_RF) offers valuable insights into tumor aggressiveness, helping oncologists tailor treatments to individual patients. Furthermore, integrating these features into predictive models can enhance prognostic tools, allowing clinicians to forecast disease progression and survival outcomes with greater accuracy, thereby improving decision-making. The RW-ensemble segmentation method, coupled with robust feature validation, addresses challenges in radiomic variability and offers a reliable pathway for incorporating these insights into routine clinical workflows, advancing precision medicine, and improving the efficiency and effectiveness of lung cancer management.

## 4. Discussion

This study highlights the transformative potential of radiomics in precision medicine, focusing on lung adenocarcinoma. The use of the RW-ensemble segmentation method for feature extraction and validation against ground-truth data revealed significant insights into the reliability and clinical utility of radiomic features. The 38 features analyzed, including GCM_RF, GRLM_RF, and histogram-based metrics, demonstrated varying degrees of correlation with ground-truth annotations, with several features exhibiting moderate to strong monotonic relationships.

This study offers valuable insights into the clinical applications of radiomics in lung cancer; however, several methodological limitations require further attention to enhance the reliability and clinical integration of the findings. One important aspect for future enhancement is the expansion beyond a single dataset. While the current dataset provides a solid foundation for methodological validation, incorporating additional datasets from diverse patient populations and imaging modalities will offer greater variability and enable more robust external validation. This broader approach will help improve model generalizability and further strengthen the clinical relevance of the findings. To address future enhancements, it should incorporate a broader range of datasets from multiple institutions, thereby ensuring a more comprehensive representation of diverse patient characteristics and imaging protocols.

The validated features identified in this study—such as skewness (histogram), convexity (GCM_RF), and high grey-level run emphasis (GRLM_RF)—offer their value as biomarkers for tumor characterization and promising opportunities for integration into existing clinical decision-making models. These features can be used to enhance early diagnostic tools, personalize treatment planning, and predict patient outcomes more accurately. For example, integrating these radiomic features into established diagnostic workflows, such as CT-based tumor evaluation or automated nodule detection systems, could streamline clinical decision-making and improve early detection. Furthermore, these features could be incorporated into prognostic models, offering oncologists a more accurate method for predicting disease progression and survival, leading to more personalized care. The RW-ensemble segmentation method can be integrated into clinical workflows as a semi-automated tool within radiology software, offering consistent and accurate delineation of complex lung nodules like juxta-pleural and juxta-vascular types. The validated features it extracts—such as convexity, skewness, and grey-level run emphasis—can support diagnostic decision systems by aiding in early malignancy detection and personalized treatment planning, thereby reducing the need for invasive procedures.

While Spearman’s correlation coefficient (SCC) is effective in assessing monotonic relationships between extracted and ground-truth features, it may not fully capture complex non-linear interactions. Future analyses will consider complementary validation metrics, such as mutual information or distance correlation, to provide a more comprehensive assessment of feature alignment.

Another critical aspect of radiomic research is its reproducibility across institutions and settings. Variability in imaging protocols, machine settings, and feature extraction methods often leads to inconsistent results, limiting the clinical applicability of radiomics. To address these challenges, the development of standardized imaging protocols and radiomics pipelines is essential. Multi-center collaborations should focus on establishing universal radiomics standards that ensure consistent data collection, feature extraction, and validation across institutions. Additionally, leveraging machine learning models for feature selection and validation could help minimize subjectivity and improve reproducibility, making radiomic analyses more robust and widely applicable in diverse clinical settings.

While the study successfully validated essential features, it also emphasizes persistent challenges in radiomics, including variability in imaging protocols, dependency on manual annotations, and the need for standardization. The integration of advanced segmentation techniques like the RW-ensemble method robustly segments complex nodule structures, underscoring its clinical relevance in handling diverse patient data. However, the reproducibility of results across institutions and populations remains a challenge, necessitating collaborative efforts for standardization.

To contextualise the performance of the proposed RW-ensemble segmentation method, we conducted a qualitative comparison with other established segmentation techniques, such as region-growing and deep learning-based models. Traditional region-growing methods, while simple and widely used, often struggle with irregular or complex nodule boundaries, particularly in juxta-pleural and juxta-vascular regions. Deep learning approaches, though powerful, typically require large, annotated datasets and may exhibit variability in performance across imaging protocols. In contrast, our RW-ensemble technique effectively balances automation with robustness, offering enhanced delineation accuracy even for anatomically challenging nodules. Table 8 summarizes the comparative characteristics, highlighting the strengths of the RW-ensemble method in terms of consistency, segmentation accuracy, and correlation-based validation. While this method demonstrates improved performance, it still relies on manually annotated ground-truth segmentations, which may introduce observer bias. Implementing fully automated segmentation techniques, such as deep learning-based methods, in future work could reduce human error and further enhance consistency across diverse cases. We can also plan to benchmark the RW-ensemble method against U-Net and other state-of-the-art deep learning architectures using publicly available datasets such as LIDC-IDRI.

Future research should focus on incorporating advanced machine learning models for automated feature selection and exploring the application of these methods across larger and more diverse datasets. Standardized pipelines and automated systems will be crucial to integrating radiomics seamlessly into clinical workflows.

## 5. Future Scope

Radiomics offers significant potential for advancing precision medicine, with several promising future directions. Integrating advanced machine learning techniques can automate segmentation and feature extraction, reducing reliance on manual processes and improving predictive accuracy. In particular, methods such as LASSO regression, principal component analysis (PCA), and minimum redundancy maximum relevance (mRMR) will be explored to reduce feature redundancy and improve interpretability. As part of our future work, we plan to incorporate external validation using larger and more diverse datasets from various institutions, which will enhance the reproducibility and generalizability of our findings. We also intend to define threshold-based criteria (e.g., SCC ≥ 0.70) for identifying radiomic features with high clinical reliability, thereby strengthening their potential as diagnostic biomarkers. The exploration of temporal radiomics (delta-radiomics) can provide insights into tumor evolution and treatment response, guiding adaptive therapies. Extending the application of validated methodologies to other cancer types and combining radiomic data with genomic, proteomic, and metabolomic information could yield comprehensive hybrid biomarkers. These advancements, coupled with seamless integration into clinical workflows, will enable more personalized, accurate, and effective diagnostic and therapeutic strategies.

## 6. Conclusions

This study demonstrates the effectiveness of the RW-ensemble segmentation method in extracting and validating radiomic features from CT scans of lung adenocarcinoma. Unlike traditional approaches, the RW-ensemble method refines segmentation across multiple slices, enhancing accuracy and robustness, particularly for complex nodule structures such as juxta-pleural and juxta-vascular nodules. The validated radiomic features, including convexity, skewness, and high grey-level run emphasis, demonstrated a strong correlation with the ground-truth data, supporting their reliability and potential as clinical biomarkers.

These findings carry meaningful clinical implications. For instance, features such as skewness and convexity may help differentiate malignant from benign nodules in early diagnosis, thereby reducing the need for invasive procedures. Additionally, these features could be integrated into existing clinical decision-making models, such as automated CT-based nodule detection systems, enabling more tailored and accurate treatment planning. Their inclusion in prognostic models also holds promise for improving survival prediction and guiding therapy adjustments.

The study’s contributions to addressing radiomic challenges, such as segmentation variability and reproducibility, provide a solid foundation for advancing radiomics as a cornerstone of precision medicine. However, broader applicability will require further efforts in standardizing imaging protocols and feature extraction methods across institutions. With these advancements, radiomics can significantly enhance lung cancer diagnosis and treatment, promoting more effective and personalized patient care.

## Figures and Tables

**Table 1:** Summary of related radiomics studies on lung cancer imaging.

Reference	Dataset	Methodology	Results	Limitations
[31]	351 patients	Developed a radiomic nomogram integrating 2D and 3D CT features	Achieved a concordance index of 0.742 for survival prediction	High computational complexity
[33]	205 PET-CT fused images	Extracted 216 wavelet-based radiomic features	Demonstrated custom segmentation accuracy	Limited to a specific segmentation approach
[18]	17 patients	Analyzed feature variability using Gini’s coefficient	Emphasized the robustness of selected features	Small patient dataset and lack of broader validation
[34]	The ITK-Snap tool segmented the dataset	Identified 10 features to classify malignant vs. benign lung nodules	Provided a manual approach for feature extraction	Manual segmentation introduces observer bias
[35]	Lung nodules dataset	Identified three features for distinguishing COVID-19 vs. benign and malignant lung nodules	Highlighted specific radiomic signatures for COVID-19 nodules	Lack of large-scale validation
[36]	309 patients	Classified histological types and cancer staging in NSCLC	Extracted 107 features for classification	Did not address real-world applicability
[37]	SCLC dataset (200 patients)	Selected nine features using LASSO for predictive modelling	Enhanced predictive accuracy for small cell lung cancer	Computational intensity of feature selection
[38]	1562 features from 309 patients		Improved risk prediction accuracy	Limited scope to specific clinical scenarios

**Table 3:** Summary of the key metrics of categorization of radiomic features.

Feature Category	Key Metrics	Description
Texture Features	Autocorrelation, Contrast, Dissimilarity, Energy, Entropy, Homogeneity	Capture local/global texture variations, uniformity, randomness, and similarity among pixel intensities.
Cluster-Based Features	Cluster Prominence, Cluster Shade	Measure skewness and asymmetry in the intensity distribution.
Shape Features	Convexity	Assess the smoothness and regularity of shapes.
Information Correlation	Information Measures of Correlation 1 and 2	Quantify dependency between neighboring pixel intensities.
Sum/Difference Metrics	Sum Average, Sum Variance, Difference Average, Difference Variance	Measure variation in pixel sums or differences.
Grey-Level Run Length Matrix	Short Run Emphasis, Long Run Emphasis, Run Percentage, Non-Uniformity (Grey Level and Run Length)	Characterize the distribution of continuous intensity runs across the image.
Histogram-Based Features	Mean, Median, Maximum, Minimum, Standard Deviation, Range, Skewness, Kurtosis, Histogram Entropy, Energy	Provide statistical summaries of intensity distributions.

**Table 4:** The strengths of SCC for positive monotonic relationships [42].

SCC Value Range	Strength
0	No correlation
0.01–0.49	Weak positive correlation
0.50–0.69	Moderate positive correlation
0.70–0.09	Strong positive correlation

**Table 5:** SCC values for selected GCM_RF features and their correlation strength.

S.No	Grey-Level Co-Occurrence Matrix (GCM_FC)	Spearman’s Correlation Coefficient (SCC)	Strength
1	gcm_rxx	0.57	Moderate
2	gcm_corr	0.44	Weak
3	gcm_conv	0.73	Strong
4	gcm_contr	0.14	Weak
5	gcm_disim	0.38	Weak
6	gcm_ener	0.50	Moderate
7	gcm_entro	0.57	Moderate
8	gcm_homo	0.35	Weak
9	gcm_imc1	0.65	Moderate
10	gcm_imc2	0.55	Moderate
11	gcm_imn	0.43	Weak
12	gcm_max_prob	0.50	Moderate

**Table 6:** SCC values for GRLM_RF features and corresponding correlation strength.

S.No	(GRLM_RF)	Spearman’s Correlation Coefficient (SCC)	Strength
1	grlm_sr	0.44	Weak
2	grlm_lr	0.86	Strong
3	grlm_gn	0.83	Strong
4	grlm_rln	0.36	Weak
5	grlm_rper	0.67	Moderate
6	grlm_hgr	0.82	Strong

**Table 7:** SCC values for histogram-based features and corresponding correlation strength.

S.No	Histogram-Based Features	Spearman’s Correlation Coefficient (SCC)	Strength
1	hist_mean	0.78	Strong
2	hist_var	0.65	Moderate
3	hist_skew	0.83	Strong
4	hist_kurt	0.37	Weak
5	hist_ener	0.77	Strong
6	hist_entro	−0.17	Strong

**Table 8:** Comparative analysis of current study with existing research.

Aspect	Current Study	Existing Research
Feature Validation	Utilizes Spearman’s Correlation Coefficient (SCC) to assess monotonic relationships, identifying strongly correlated features like skewness (SCC = 0.83) and high grey-level run emphasis (SCC = 0.82).	Studies such as Aerts et al. [26] emphasize the predictive power of texture features, aligning with findings on skewness and high grey-level run emphasis for survival analysis.
Segmentation Method	Employs RW-ensemble segmentation to improve accuracy for complex nodule types (juxta-pleural and juxta-vascular), reducing observer variability.	Earlier works like Ganeshan et al. [9] and Tamponi et al. [18] relied on manual or neural network-based segmentation, which faced challenges with observer bias and initialization.
Key Shape Feature	Convexity (GCM_RF, SCC = 0.73) is highlighted for its role in tumor geometry and progression.	Consistent with Huang et al. [32], which underscores the clinical relevance of shape-based features in evaluating tumor invasiveness.
Feature Overlap	Weak correlations for features like kurtosis (SCC = 0.37) and dissimilarity (SCC = 0.38), potentially due to redundancy with other metrics.	Overlap in feature utility, as seen in studies such as Parmar et al. [25], indicates challenges in isolating independent contributions of specific features.
Clinical Implications	Strongly correlated features support early differentiation of malignant vs. benign nodules, reducing invasive procedures. Convexity aids in predicting invasiveness and planning treatments.	Similar implications are noted in works like Aerts et al. [26] emphasizing the utility of validated features for survival analysis and precision oncology.
Methodological Advances	Introduces RW-ensemble segmentation to overcome limitations of manual and single-modality approaches.	Previous studies primarily relied on traditional segmentation or single-modality methods, often lacking robustness and generalizability (e.g., Parmar et al. [25]).
Limitations	Limited dataset diversity and potential feature redundancy impact weak correlations.	Dataset variability and feature standardization challenges are common themes in prior research, as noted by Ganeshan et al. [9] and Aerts et al. [26].

## Data Availability

The dataset was collected from the TCIA consortium https://www.cancerimagingarchive.net/collection/lungct-diagnosis/ (accessed on 30 December 2014).
